# A second monoclinic polymorph of 4-[(*E*)-(4-benzyl­oxybenzyl­idene)amino]-1,5-dimethyl-2-phenyl-1*H*-pyrazol-3(2*H*)-one

**DOI:** 10.1107/S1600536812050891

**Published:** 2012-12-19

**Authors:** Rajni Kant, Vivek K. Gupta, Kamini Kapoor, Prakash S. Nayak, B. K. Sarojini, B. Narayana

**Affiliations:** a X-ray Crystallography Laboratory, Post-Graduate Department of Physics & Electronics, University of Jammu, Jammu Tawi 180 006, India; b Department of Studies in Chemistry, Mangalore University, Mangalagangotri 574 199, India; c Department of Chemistry, P.A. College of Engineering, Nadupadavu, Mangalore 574 153, India

## Abstract

In the title compound, C_25_H_23_N_3_O_2_, the central benzene ring makes dihedral angles of 77.14 (8) and 87.7 (2)° with the terminal benzene rings and an angle of 1.9 (1)° with the pyrazolone ring. The benzene ring and the N atom of the pyrazole ring bearing the phenyl substituent are disordered over two sets of sites with an occupancy ratio of 0.71 (2):0.29 (2). The N atoms of the pyrazole ring have a pyramidal environment, the sums of the valence angles around them being 354.6 (3) and 352.0 (6)/349.5 (15)°. In the crystal, mol­ecules are packed into layers parallel to the *ac* plane. The other monoclinic polymorphic form was reported recently [Dutkiewicz *et al.* (2012 ▶). *Acta Cryst.* E**68**, o1324].

## Related literature

For potential applications of Schiff bases, see: Patole *et al.* (2006 ▶); Shi *et al.*(2007 ▶); Satyanarayana *et al.* (2008 ▶). For related structures, see: Liu *et al.*(2008 ▶); Hu, (2006 ▶). For the other monoclinic polymorph, see Dutkiewicz *et al.*, (2012 ▶).
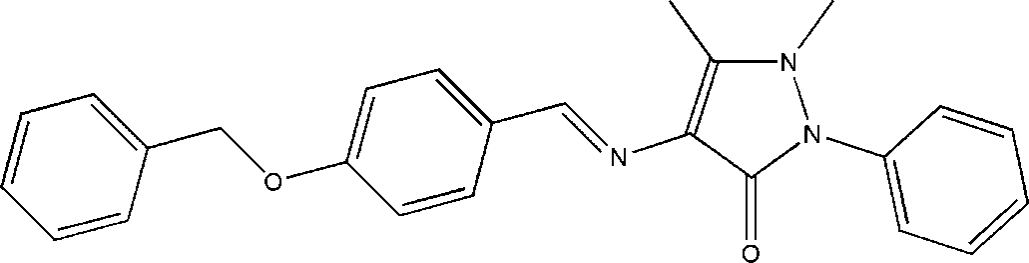


## Experimental

### 

#### Crystal data

C_25_H_23_N_3_O_2_
*M* *_r_* = 397.46
Monoclinic, 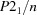

*a* = 13.0079 (5) Å
*b* = 9.9079 (4) Å
*c* = 17.0469 (9) Å
β = 103.674 (4)°
*V* = 2134.75 (16) Å^3^
*Z* = 4
Mo *K*α radiation
μ = 0.08 mm^−1^
*T* = 293 K
0.3 × 0.2 × 0.2 mm

#### Data collection

Oxford Diffraction Xcalibur Sapphire3 diffractometer
Absorption correction: multi-scan (*CrysAlis PRO*; Oxford Diffraction, 2010 ▶) *T*_min_ = 0.920, *T*_max_ = 1.000
19519 measured reflections
4162 independent reflections
2399 reflections with *I* > 2σ(*I*)
*R*_int_ = 0.047

#### Refinement

*R*[*F* ^2^ > 2σ(*F* ^2^)] = 0.055
*wR*(*F* ^2^) = 0.154
*S* = 1.03
4162 reflections
292 parameters
12 restraints
H-atom parameters constrained
Δρ_max_ = 0.21 e Å^−3^
Δρ_min_ = −0.15 e Å^−3^

### 

Data collection: *CrysAlis PRO* (Oxford Diffraction, 2010 ▶); cell refinement: *CrysAlis PRO*; data reduction: *CrysAlis PRO*; program(s) used to solve structure: *SHELXS97* (Sheldrick, 2008 ▶); program(s) used to refine structure: *SHELXL97* (Sheldrick, 2008 ▶); molecular graphics: *ORTEP-3* (Farrugia, 2012) ▶; software used to prepare material for publication: *PLATON* (Spek, 2009 ▶).

## Supplementary Material

Crystal structure: contains datablock(s) I, global. DOI: 10.1107/S1600536812050891/gk2544sup1.cif

Structure factors: contains datablock(s) I. DOI: 10.1107/S1600536812050891/gk2544Isup2.hkl

Supplementary material file. DOI: 10.1107/S1600536812050891/gk2544Isup3.cml

Additional supplementary materials:  crystallographic information; 3D view; checkCIF report

## Figures and Tables

**Fig. 1.**
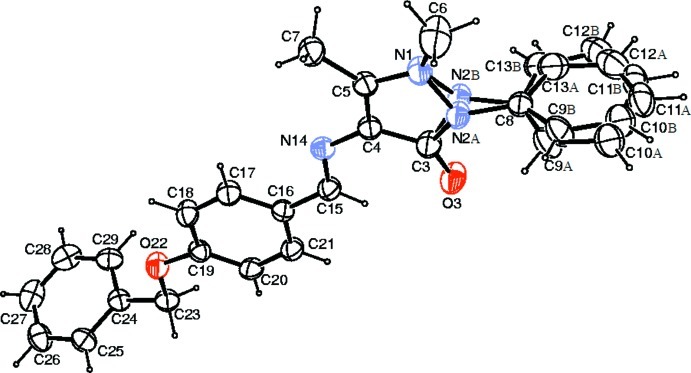
ORTEP view of the molecule with the atom-labeling scheme. The displacement ellipsoids are drawn at the 40% probability level. H atoms are shown as small spheres of arbitrary radii.

**Fig. 2.**
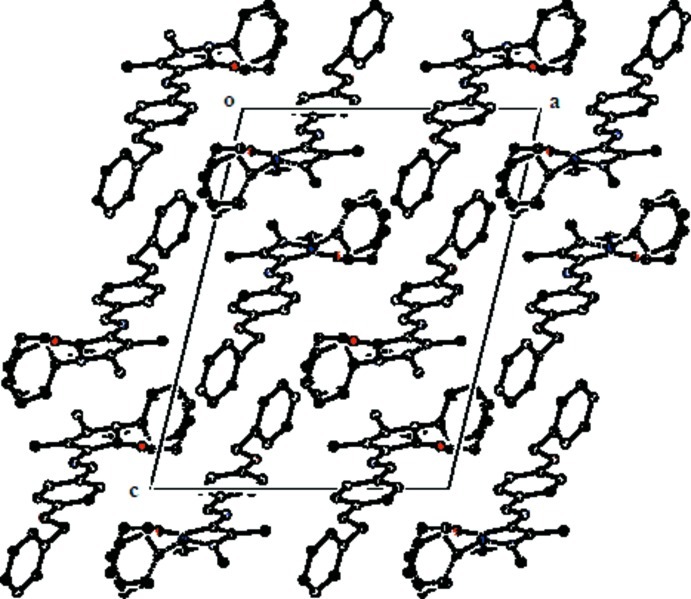
The packing arrangement of molecules viewed down the b axis. Hydrogen atoms have been omitted for clarity.

## supplementary crystallographic information

### Comment

Schiff bases are widely used organic compounds and structurally it is a nitrogen analogue of an aldehyde or ketone in which the carbonyl group has been replaced by an imine or azomethine group. They are used as pigments and dyes, catalysts, intermediates in organic synthesis, and polymer stabilisers. Schiff bases have also been shown to exhibit a broad range of biological activities including antimicrobial (Shi *et al.*, 2007; Satyanarayana *et al.*, 2008; antimycobacterial (Patole *et al.*, 2006). In view of the pharmacological importance of schiff base derivatives, the title compound (I) is prepared and its crystal structure is reported.

All bond lengths and angles are normal and correspond to those observed in related structures(Liu *et al.*, 2008; Hu, 2006; Dutkiewicz *et al.*, 2012). The central benzene ring makes dihedral angles of 77.14 (8), 87.7 (2) and 87.1 (6)° with the terminal benzene rings (C24—C29),(C8—C13A)and (C8—C13B) respectively while 1.9 (1)(N2A)/ 1.7 (1)°(N2B) with the pyrazolone ring. The benzene ring (C8—C13) and atom N2 are disordered over two positions with an occupancy ratio of 0.71 (2):0.29 (2). The N atoms of the pyrazole ring have a pyramidal environment, the sums of the valence angles around them being 354.6 (3)(N1) and 352.0 (6)(N2A) / 349.5 (15)(N2B) °. The sums of the valence angles around N-atoms of the pyrazole ring in the polymorph of this compound are 353.5 for N1 and 347.3 ° for N2 (Dutkiewicz *et al.*, 2012). Molecules are packed into layers parallel to the *ac*-plane (Fig.2).

### Experimental

The title compound was synthesized by adding 4-benzyloxybenzaldehyde (0.212 g, 1 mmol) to the solution of 4-aminoantipyrine (0.203 g, 1 mmol) in methanol (30 ml) containing few drops of conc. sulfuric acid. The mixture was refluxed for 3hrs and left stirring overnight at room temperature. The resultant solid obtained was then filtered. Yellow needle-shaped single crystals suitable for X-ray structure determination were formed after slow evaporation of dichloromethane at room temperature (431–433 K).

### Refinement

Atom N2 and the benzene ring (C8—C13) are disordered over two positions with an occupancy ratio of 0.71 (2):0.29 (2). In the refinement process restraints were imposed on C—C [1.38 (1) Å] distances of the disordered molecular fragments and the displacement parameters. All H atoms were positioned geometrically and were treated as riding on their parent C atoms, with C—H distances of 0.93–0.97 Å and with *U*_iso_(H) = 1.2*U*_eq_(C) or 1.5*U*_eq_(methyl C).

### Crystal data

**Table:**

C_25_H_23_N_3_O_2_	*F*(000) = 840
*M**_r_* = 397.46	*D*_x_ = 1.237 Mg m^−^^3^
Monoclinic, *P*2_1_/*n*	Mo *K*α radiation, λ = 0.71073 Å
Hall symbol: -P 2yn	Cell parameters from 7057 reflections
*a* = 13.0079 (5) Å	θ = 3.6–29.0°
*b* = 9.9079 (4) Å	µ = 0.08 mm^−^^1^
*c* = 17.0469 (9) Å	*T* = 293 K
β = 103.674 (4)°	Plate, white
*V* = 2134.75 (16) Å^3^	0.3 × 0.2 × 0.2 mm
*Z* = 4	

### Data collection

**Table:**

Oxford Diffraction Xcalibur Sapphire3 diffractometer	4162 independent reflections
Radiation source: fine-focus sealed tube	2399 reflections with *I* > 2σ(*I*)
Graphite monochromator	*R*_int_ = 0.047
Detector resolution: 16.1049 pixels mm^-1^	θ_max_ = 26.0°, θ_min_ = 3.6°
ω scans	*h* = −15→16
Absorption correction: multi-scan (*CrysAlis PRO*; Oxford Diffraction, 2010)	*k* = −12→12
*T*_min_ = 0.920, *T*_max_ = 1.000	*l* = −21→21
19519 measured reflections	

### Refinement

**Table:**

Refinement on *F*^2^	Primary atom site location: structure-invariant direct methods
Least-squares matrix: full	Secondary atom site location: difference Fourier map
*R*[*F*^2^ > 2σ(*F*^2^)] = 0.055	Hydrogen site location: inferred from neighbouring sites
*wR*(*F*^2^) = 0.154	H-atom parameters constrained
*S* = 1.03	*w* = 1/[σ^2^(*F*_o_^2^) + (0.057*P*)^2^ + 0.3022*P*] where *P* = (*F*_o_^2^ + 2*F*_c_^2^)/3
4162 reflections	(Δ/σ)_max_ = 0.001
292 parameters	Δρ_max_ = 0.21 e Å^−^^3^
12 restraints	Δρ_min_ = −0.15 e Å^−^^3^

### Special details

**Table:**

Experimental. *CrysAlis PRO*, Oxford Diffraction Ltd., Version 1.171.34.40 (release 27–08-2010 CrysAlis171. NET) (compiled Aug 27 2010,11:50:40) Empirical absorption correction using spherical harmonics, implemented in SCALE3 ABSPACK scaling algorithm.
Geometry. All e.s.d.'s (except the e.s.d. in the dihedral angle between two l.s. planes) are estimated using the full covariance matrix. The cell e.s.d.'s are taken into account individually in the estimation of e.s.d.'s in distances, angles and torsion angles; correlations between e.s.d.'s in cell parameters are only used when they are defined by crystal symmetry. An approximate (isotropic) treatment of cell e.s.d.'s is used for estimating e.s.d.'s involving l.s. planes.
Refinement. Refinement of *F*^2^ against ALL reflections. The weighted *R*-factor *wR* and goodness of fit *S* are based on *F*^2^, conventional *R*-factors *R* are based on *F*, with *F* set to zero for negative *F*^2^. The threshold expression of *F*^2^ > σ(*F*^2^) is used only for calculating *R*-factors(gt) *etc*. and is not relevant to the choice of reflections for refinement. *R*-factors based on *F*^2^ are statistically about twice as large as those based on *F*, and *R*- factors based on ALL data will be even larger.

### Fractional atomic coordinates and isotropic or equivalent isotropic displacement parameters (Å^2^)

**Table:**

	*x*	*y*	*z*	*U*_iso_*/*U*_eq_	Occ. (<1)
N1	0.74159 (14)	−0.03151 (19)	−0.14892 (13)	0.0628 (5)	
N2A	0.8373 (6)	0.0248 (9)	−0.1606 (6)	0.0613 (17)	0.707 (17)
C9A	0.9286 (11)	−0.0547 (11)	−0.2496 (4)	0.0742 (16)	0.707 (17)
H9A	0.8866	0.0057	−0.2850	0.089*	0.707 (17)
C10A	1.0017 (8)	−0.1332 (10)	−0.2750 (6)	0.091 (2)	0.707 (17)
H10A	1.0089	−0.1260	−0.3278	0.109*	0.707 (17)
C11A	1.0640 (6)	−0.2218 (8)	−0.2229 (7)	0.083 (3)	0.707 (17)
H11A	1.1114	−0.2774	−0.2408	0.100*	0.707 (17)
C12A	1.0560 (6)	−0.2277 (9)	−0.1448 (6)	0.092 (3)	0.707 (17)
H12A	1.1003	−0.2849	−0.1087	0.110*	0.707 (17)
C13A	0.9831 (12)	−0.1502 (15)	−0.1191 (5)	0.080 (2)	0.707 (17)
H13A	0.9779	−0.1548	−0.0657	0.096*	0.707 (17)
N2B	0.8462 (17)	0.001 (2)	−0.1335 (11)	0.0613 (17)	0.293 (17)
C9B	0.940 (3)	−0.089 (3)	−0.2461 (10)	0.0742 (16)	0.293 (17)
H9B	0.8994	−0.0473	−0.2919	0.089*	0.293 (17)
C10B	1.021 (2)	−0.173 (3)	−0.2531 (15)	0.091 (2)	0.293 (17)
H10B	1.0390	−0.1891	−0.3021	0.109*	0.293 (17)
C11B	1.074 (2)	−0.233 (3)	−0.1820 (14)	0.083 (3)	0.293 (17)
H11B	1.1310	−0.2881	−0.1851	0.100*	0.293 (17)
C12B	1.054 (2)	−0.220 (3)	−0.1069 (13)	0.092 (3)	0.293 (17)
H12B	1.0900	−0.2679	−0.0622	0.110*	0.293 (17)
C13B	0.974 (3)	−0.129 (4)	−0.1031 (11)	0.080 (2)	0.293 (17)
H13B	0.9589	−0.1105	−0.0537	0.096*	0.293 (17)
O3	0.93832 (13)	0.21006 (18)	−0.11143 (13)	0.0865 (6)	
C3	0.85441 (18)	0.1476 (2)	−0.12022 (15)	0.0626 (7)	
C4	0.75575 (16)	0.1790 (2)	−0.10095 (13)	0.0503 (5)	
C5	0.69005 (16)	0.0687 (2)	−0.11997 (14)	0.0530 (6)	
C6	0.6960 (2)	−0.1478 (3)	−0.1940 (2)	0.1129 (13)	
H6A	0.6383	−0.1805	−0.1731	0.169*	
H6B	0.7487	−0.2171	−0.1893	0.169*	
H6C	0.6705	−0.1238	−0.2498	0.169*	
C7	0.57995 (17)	0.0514 (3)	−0.11114 (17)	0.0737 (8)	
H7A	0.5331	0.0412	−0.1635	0.111*	
H7B	0.5596	0.1294	−0.0849	0.111*	
H7C	0.5760	−0.0274	−0.0793	0.111*	
C8	0.91815 (17)	−0.0660 (2)	−0.17219 (15)	0.0558 (6)	
N14	0.72512 (13)	0.29631 (18)	−0.06847 (11)	0.0517 (5)	
C15	0.78748 (17)	0.3983 (2)	−0.05482 (13)	0.0542 (6)	
H15	0.8538	0.3918	−0.0661	0.065*	
C16	0.75724 (16)	0.5243 (2)	−0.02202 (13)	0.0496 (5)	
C17	0.65779 (17)	0.5437 (2)	−0.00752 (14)	0.0601 (6)	
H17	0.6080	0.4748	−0.0196	0.072*	
C18	0.63120 (17)	0.6619 (2)	0.02411 (15)	0.0619 (6)	
H18	0.5636	0.6731	0.0325	0.074*	
C19	0.70499 (17)	0.7655 (2)	0.04375 (13)	0.0506 (5)	
C20	0.80368 (18)	0.7488 (2)	0.02936 (14)	0.0573 (6)	
H20	0.8534	0.8178	0.0415	0.069*	
C21	0.82894 (18)	0.6293 (2)	−0.00321 (15)	0.0588 (6)	
H21	0.8960	0.6191	−0.0128	0.071*	
O22	0.67048 (11)	0.87620 (16)	0.07728 (10)	0.0647 (5)	
C23	0.74356 (17)	0.9854 (2)	0.10298 (15)	0.0583 (6)	
H23A	0.7578	1.0319	0.0566	0.070*	
H23B	0.8098	0.9516	0.1359	0.070*	
C24	0.69226 (16)	1.0782 (2)	0.15077 (14)	0.0522 (6)	
C25	0.64419 (18)	1.1960 (2)	0.11844 (15)	0.0642 (7)	
H25	0.6458	1.2212	0.0662	0.077*	
C26	0.5933 (2)	1.2772 (3)	0.16364 (19)	0.0761 (8)	
H26	0.5612	1.3570	0.1417	0.091*	
C27	0.5903 (2)	1.2403 (3)	0.2406 (2)	0.0822 (9)	
H27	0.5551	1.2941	0.2705	0.099*	
C28	0.6390 (2)	1.1244 (3)	0.27318 (17)	0.0788 (8)	
H28	0.6375	1.0996	0.3255	0.095*	
C29	0.68990 (19)	1.0448 (3)	0.22883 (15)	0.0652 (7)	
H29	0.7236	0.9666	0.2518	0.078*	

### Atomic displacement parameters (Å^2^)

**Table:**

	*U*^11^	*U*^22^	*U*^33^	*U*^12^	*U*^13^	*U*^23^
N1	0.0479 (11)	0.0503 (12)	0.0897 (15)	−0.0096 (9)	0.0154 (9)	−0.0168 (11)
N2A	0.0523 (19)	0.052 (3)	0.085 (5)	−0.0073 (19)	0.026 (3)	−0.016 (3)
C9A	0.095 (4)	0.053 (6)	0.074 (2)	0.014 (4)	0.018 (2)	−0.011 (2)
C10A	0.120 (5)	0.069 (6)	0.094 (5)	0.005 (4)	0.047 (4)	−0.019 (3)
C11A	0.071 (3)	0.061 (3)	0.120 (8)	0.001 (2)	0.027 (5)	−0.029 (5)
C12A	0.082 (3)	0.072 (3)	0.111 (8)	0.010 (2)	0.004 (6)	0.021 (7)
C13A	0.081 (3)	0.076 (6)	0.085 (3)	−0.003 (2)	0.023 (3)	0.012 (4)
N2B	0.0523 (19)	0.052 (3)	0.085 (5)	−0.0073 (19)	0.026 (3)	−0.016 (3)
C9B	0.095 (4)	0.053 (6)	0.074 (2)	0.014 (4)	0.018 (2)	−0.011 (2)
C10B	0.120 (5)	0.069 (6)	0.094 (5)	0.005 (4)	0.047 (4)	−0.019 (3)
C11B	0.071 (3)	0.061 (3)	0.120 (8)	0.001 (2)	0.027 (5)	−0.029 (5)
C12B	0.082 (3)	0.072 (3)	0.111 (8)	0.010 (2)	0.004 (6)	0.021 (7)
C13B	0.081 (3)	0.076 (6)	0.085 (3)	−0.003 (2)	0.023 (3)	0.012 (4)
O3	0.0631 (10)	0.0667 (12)	0.1405 (17)	−0.0236 (9)	0.0459 (11)	−0.0353 (12)
C3	0.0549 (14)	0.0537 (15)	0.0823 (17)	−0.0109 (12)	0.0225 (12)	−0.0157 (13)
C4	0.0473 (12)	0.0464 (13)	0.0565 (13)	−0.0036 (10)	0.0109 (10)	−0.0041 (11)
C5	0.0475 (12)	0.0478 (13)	0.0614 (14)	−0.0043 (10)	0.0087 (10)	−0.0041 (11)
C6	0.0795 (19)	0.093 (2)	0.169 (3)	−0.0320 (17)	0.036 (2)	−0.077 (2)
C7	0.0510 (14)	0.0625 (17)	0.107 (2)	−0.0074 (12)	0.0179 (13)	−0.0091 (15)
C8	0.0524 (13)	0.0467 (13)	0.0708 (16)	−0.0081 (11)	0.0198 (11)	−0.0080 (12)
N14	0.0528 (10)	0.0448 (11)	0.0569 (11)	−0.0055 (9)	0.0118 (8)	−0.0037 (9)
C15	0.0517 (12)	0.0514 (14)	0.0620 (15)	−0.0035 (11)	0.0182 (10)	−0.0038 (11)
C16	0.0503 (12)	0.0436 (12)	0.0553 (13)	−0.0017 (10)	0.0133 (10)	−0.0004 (10)
C17	0.0502 (13)	0.0492 (14)	0.0800 (17)	−0.0103 (11)	0.0136 (11)	−0.0114 (12)
C18	0.0448 (12)	0.0567 (15)	0.0838 (17)	−0.0063 (11)	0.0144 (11)	−0.0148 (13)
C19	0.0514 (12)	0.0436 (13)	0.0556 (13)	−0.0005 (10)	0.0104 (10)	−0.0030 (10)
C20	0.0576 (14)	0.0453 (13)	0.0713 (15)	−0.0138 (11)	0.0196 (11)	−0.0047 (11)
C21	0.0562 (13)	0.0502 (14)	0.0758 (16)	−0.0067 (11)	0.0269 (11)	−0.0055 (12)
O22	0.0539 (9)	0.0510 (10)	0.0889 (12)	−0.0065 (7)	0.0160 (8)	−0.0187 (9)
C23	0.0579 (13)	0.0472 (13)	0.0692 (15)	−0.0103 (11)	0.0139 (11)	−0.0051 (12)
C24	0.0509 (12)	0.0420 (13)	0.0609 (15)	−0.0090 (10)	0.0078 (10)	−0.0097 (11)
C25	0.0730 (16)	0.0533 (15)	0.0640 (15)	0.0010 (13)	0.0114 (12)	−0.0037 (13)
C26	0.0736 (17)	0.0580 (17)	0.092 (2)	0.0069 (13)	0.0099 (15)	−0.0164 (16)
C27	0.0699 (17)	0.086 (2)	0.093 (2)	−0.0173 (16)	0.0251 (16)	−0.0460 (19)
C28	0.094 (2)	0.082 (2)	0.0629 (17)	−0.0235 (18)	0.0235 (15)	−0.0181 (16)
C29	0.0727 (15)	0.0585 (16)	0.0612 (16)	−0.0133 (13)	0.0093 (12)	−0.0018 (13)

### Geometric parameters (Å, º)

**Table:**

N1—C5	1.355 (3)	C6—H6A	0.9600
N1—N2B	1.36 (2)	C6—H6B	0.9600
N1—N2A	1.421 (8)	C6—H6C	0.9600
N1—C6	1.434 (3)	C7—H7A	0.9600
N2A—C3	1.390 (8)	C7—H7B	0.9600
N2A—C8	1.433 (9)	C7—H7C	0.9600
C9A—C8	1.363 (5)	N14—C15	1.282 (3)
C9A—C10A	1.375 (6)	C15—C16	1.459 (3)
C9A—H9A	0.9300	C15—H15	0.9300
C10A—C11A	1.369 (6)	C16—C21	1.384 (3)
C10A—H10A	0.9300	C16—C17	1.387 (3)
C11A—C12A	1.361 (6)	C17—C18	1.367 (3)
C11A—H11A	0.9300	C17—H17	0.9300
C12A—C13A	1.370 (6)	C18—C19	1.391 (3)
C12A—H12A	0.9300	C18—H18	0.9300
C13A—C8	1.366 (5)	C19—O22	1.361 (2)
C13A—H13A	0.9300	C19—C20	1.373 (3)
N2B—C8	1.43 (2)	C20—C21	1.380 (3)
N2B—C3	1.47 (2)	C20—H20	0.9300
C9B—C10B	1.373 (10)	C21—H21	0.9300
C9B—C8	1.376 (10)	O22—C23	1.438 (2)
C9B—H9B	0.9300	C23—C24	1.487 (3)
C10B—C11B	1.377 (10)	C23—H23A	0.9700
C10B—H10B	0.9300	C23—H23B	0.9700
C11B—C12B	1.375 (10)	C24—C25	1.376 (3)
C11B—H11B	0.9300	C24—C29	1.379 (3)
C12B—C13B	1.377 (10)	C25—C26	1.385 (3)
C12B—H12B	0.9300	C25—H25	0.9300
C13B—C8	1.381 (10)	C26—C27	1.371 (4)
C13B—H13B	0.9300	C26—H26	0.9300
O3—C3	1.233 (2)	C27—C28	1.364 (4)
C3—C4	1.432 (3)	C27—H27	0.9300
C4—C5	1.378 (3)	C28—C29	1.367 (4)
C4—N14	1.386 (3)	C28—H28	0.9300
C5—C7	1.485 (3)	C29—H29	0.9300
			
C5—N1—N2B	108.1 (10)	H7A—C7—H7C	109.5
C5—N1—N2A	106.8 (4)	H7B—C7—H7C	109.5
C5—N1—C6	127.36 (19)	C9A—C8—C13A	120.3 (5)
N2B—N1—C6	124.3 (10)	C13A—C8—C9B	106.0 (10)
N2A—N1—C6	120.4 (4)	C9A—C8—C13B	135.1 (12)
C3—N2A—N1	108.5 (6)	C9B—C8—C13B	121.5 (14)
C3—N2A—C8	125.5 (6)	C9A—C8—N2B	129.3 (9)
N1—N2A—C8	118.0 (6)	C13A—C8—N2B	110.4 (10)
C8—C9A—C10A	119.5 (7)	C9B—C8—N2B	143.1 (14)
C8—C9A—H9A	120.2	C13B—C8—N2B	95.2 (14)
C10A—C9A—H9A	120.2	C9A—C8—N2A	109.0 (7)
C11A—C10A—C9A	120.4 (7)	C13A—C8—N2A	130.6 (8)
C11A—C10A—H10A	119.8	C9B—C8—N2A	123.2 (12)
C9A—C10A—H10A	119.8	C13B—C8—N2A	115.2 (12)
C12A—C11A—C10A	119.4 (6)	C15—N14—C4	120.29 (18)
C12A—C11A—H11A	120.3	N14—C15—C16	121.8 (2)
C10A—C11A—H11A	120.3	N14—C15—H15	119.1
C11A—C12A—C13A	120.5 (6)	C16—C15—H15	119.1
C11A—C12A—H12A	119.7	C21—C16—C17	117.4 (2)
C13A—C12A—H12A	119.7	C21—C16—C15	120.27 (19)
C8—C13A—C12A	119.7 (7)	C17—C16—C15	122.3 (2)
C8—C13A—H13A	120.1	C18—C17—C16	121.5 (2)
C12A—C13A—H13A	120.1	C18—C17—H17	119.2
N1—N2B—C8	122.0 (16)	C16—C17—H17	119.2
N1—N2B—C3	107.4 (15)	C17—C18—C19	120.1 (2)
C8—N2B—C3	120.1 (14)	C17—C18—H18	119.9
C10B—C9B—C8	120.7 (19)	C19—C18—H18	119.9
C10B—C9B—H9B	119.6	O22—C19—C20	126.0 (2)
C8—C9B—H9B	119.6	O22—C19—C18	114.73 (19)
C9B—C10B—C11B	115 (2)	C20—C19—C18	119.3 (2)
C9B—C10B—H10B	122.6	C19—C20—C21	119.8 (2)
C11B—C10B—H10B	122.6	C19—C20—H20	120.1
C12B—C11B—C10B	128 (2)	C21—C20—H20	120.1
C12B—C11B—H11B	116.2	C20—C21—C16	121.8 (2)
C10B—C11B—H11B	116.2	C20—C21—H21	119.1
C11B—C12B—C13B	115 (2)	C16—C21—H21	119.1
C11B—C12B—H12B	122.6	C19—O22—C23	118.30 (16)
C13B—C12B—H12B	122.6	O22—C23—C24	106.39 (16)
C12B—C13B—C8	120 (2)	O22—C23—H23A	110.5
C12B—C13B—H13B	119.8	C24—C23—H23A	110.5
C8—C13B—H13B	119.8	O22—C23—H23B	110.5
O3—C3—N2A	122.2 (4)	C24—C23—H23B	110.5
O3—C3—C4	132.8 (2)	H23A—C23—H23B	108.6
N2A—C3—C4	104.9 (4)	C25—C24—C29	118.6 (2)
O3—C3—N2B	123.0 (9)	C25—C24—C23	121.7 (2)
C4—C3—N2B	102.3 (9)	C29—C24—C23	119.7 (2)
C5—C4—N14	122.57 (19)	C24—C25—C26	120.1 (2)
C5—C4—C3	108.27 (19)	C24—C25—H25	120.0
N14—C4—C3	129.16 (19)	C26—C25—H25	120.0
N1—C5—C4	109.51 (18)	C27—C26—C25	120.2 (3)
N1—C5—C7	121.67 (19)	C27—C26—H26	119.9
C4—C5—C7	128.8 (2)	C25—C26—H26	119.9
N1—C6—H6A	109.5	C28—C27—C26	119.9 (3)
N1—C6—H6B	109.5	C28—C27—H27	120.0
H6A—C6—H6B	109.5	C26—C27—H27	120.0
N1—C6—H6C	109.5	C27—C28—C29	119.9 (3)
H6A—C6—H6C	109.5	C27—C28—H28	120.0
H6B—C6—H6C	109.5	C29—C28—H28	120.0
C5—C7—H7A	109.5	C28—C29—C24	121.3 (3)
C5—C7—H7B	109.5	C28—C29—H29	119.3
H7A—C7—H7B	109.5	C24—C29—H29	119.3
C5—C7—H7C	109.5		
			
C5—N1—N2A—C3	14.6 (7)	C10B—C9B—C8—C9A	152 (12)
N2B—N1—N2A—C3	−82 (4)	C10B—C9B—C8—C13A	−6 (4)
C6—N1—N2A—C3	170.3 (4)	C10B—C9B—C8—C13B	−2 (5)
C5—N1—N2A—C8	165.2 (5)	C10B—C9B—C8—N2B	−176 (2)
N2B—N1—N2A—C8	68 (3)	C10B—C9B—C8—N2A	177 (2)
C6—N1—N2A—C8	−39.1 (9)	C12B—C13B—C8—C9A	−11 (6)
C8—C9A—C10A—C11A	−0.3 (18)	C12B—C13B—C8—C13A	12 (7)
C9A—C10A—C11A—C12A	−2.6 (16)	C12B—C13B—C8—C9B	−1 (6)
C10A—C11A—C12A—C13A	2.8 (16)	C12B—C13B—C8—N2B	175 (4)
C11A—C12A—C13A—C8	0 (2)	C12B—C13B—C8—N2A	179 (3)
C5—N1—N2B—C8	−165.2 (11)	N1—N2B—C8—C9A	74 (2)
N2A—N1—N2B—C8	−76 (3)	C3—N2B—C8—C9A	−66.3 (19)
C6—N1—N2B—C8	10.3 (18)	N1—N2B—C8—C13A	−106.7 (16)
C5—N1—N2B—C3	−20.5 (12)	C3—N2B—C8—C13A	112.8 (14)
N2A—N1—N2B—C3	69 (3)	N1—N2B—C8—C9B	63 (4)
C6—N1—N2B—C3	155.0 (7)	C3—N2B—C8—C9B	−77 (3)
C8—C9B—C10B—C11B	2 (5)	N1—N2B—C8—C13B	−112 (3)
C9B—C10B—C11B—C12B	2 (6)	C3—N2B—C8—C13B	108 (2)
C10B—C11B—C12B—C13B	−5 (6)	N1—N2B—C8—N2A	79 (3)
C11B—C12B—C13B—C8	4 (6)	C3—N2B—C8—N2A	−62 (3)
N1—N2A—C3—O3	170.4 (4)	C3—N2A—C8—C9A	−103.3 (10)
C8—N2A—C3—O3	22.6 (10)	N1—N2A—C8—C9A	111.6 (10)
N1—N2A—C3—C4	−13.6 (7)	C3—N2A—C8—C13A	73.6 (14)
C8—N2A—C3—C4	−161.4 (7)	N1—N2A—C8—C13A	−71.5 (13)
N1—N2A—C3—N2B	72 (3)	C3—N2A—C8—C9B	−110 (2)
C8—N2A—C3—N2B	−76 (3)	N1—N2A—C8—C9B	104 (2)
N1—N2B—C3—O3	−173.8 (6)	C3—N2A—C8—C13B	69 (3)
C8—N2B—C3—O3	−28.3 (18)	N1—N2A—C8—C13B	−76 (3)
N1—N2B—C3—N2A	−80 (3)	C3—N2A—C8—N2B	80 (3)
C8—N2B—C3—N2A	66 (3)	N1—N2A—C8—N2B	−65 (3)
N1—N2B—C3—C4	20.1 (12)	C5—C4—N14—C15	−177.2 (2)
C8—N2B—C3—C4	165.6 (12)	C3—C4—N14—C15	3.2 (4)
O3—C3—C4—C5	−176.7 (3)	C4—N14—C15—C16	178.91 (19)
N2A—C3—C4—C5	8.0 (5)	N14—C15—C16—C21	175.9 (2)
N2B—C3—C4—C5	−12.6 (8)	N14—C15—C16—C17	−3.4 (3)
O3—C3—C4—N14	3.0 (5)	C21—C16—C17—C18	0.0 (4)
N2A—C3—C4—N14	−172.3 (5)	C15—C16—C17—C18	179.3 (2)
N2B—C3—C4—N14	167.1 (8)	C16—C17—C18—C19	−1.0 (4)
N2B—N1—C5—C4	12.4 (8)	C17—C18—C19—O22	−177.9 (2)
N2A—N1—C5—C4	−9.4 (5)	C17—C18—C19—C20	1.5 (4)
C6—N1—C5—C4	−162.9 (3)	O22—C19—C20—C21	178.3 (2)
N2B—N1—C5—C7	−166.7 (8)	C18—C19—C20—C21	−0.9 (4)
N2A—N1—C5—C7	171.5 (4)	C19—C20—C21—C16	−0.1 (4)
C6—N1—C5—C7	18.0 (4)	C17—C16—C21—C20	0.5 (3)
N14—C4—C5—N1	−178.8 (2)	C15—C16—C21—C20	−178.7 (2)
C3—C4—C5—N1	1.0 (3)	C20—C19—O22—C23	−2.2 (3)
N14—C4—C5—C7	0.3 (4)	C18—C19—O22—C23	177.04 (19)
C3—C4—C5—C7	180.0 (2)	C19—O22—C23—C24	−169.24 (18)
C10A—C9A—C8—C13A	3.0 (18)	O22—C23—C24—C25	−101.5 (2)
C10A—C9A—C8—C9B	−22 (8)	O22—C23—C24—C29	76.8 (2)
C10A—C9A—C8—C13B	10 (3)	C29—C24—C25—C26	−1.0 (3)
C10A—C9A—C8—N2B	−178.0 (14)	C23—C24—C25—C26	177.3 (2)
C10A—C9A—C8—N2A	−179.7 (10)	C24—C25—C26—C27	−0.3 (4)
C12A—C13A—C8—C9A	−3 (2)	C25—C26—C27—C28	1.1 (4)
C12A—C13A—C8—C9B	4 (2)	C26—C27—C28—C29	−0.5 (4)
C12A—C13A—C8—C13B	−164 (12)	C27—C28—C29—C24	−0.8 (4)
C12A—C13A—C8—N2B	178.0 (15)	C25—C24—C29—C28	1.6 (3)
C12A—C13A—C8—N2A	−179.5 (9)	C23—C24—C29—C28	−176.8 (2)
